# Hypnosis Produced in Battle

**Published:** 1915-04-10

**Authors:** 


### HYPNOSIS PRODUCED IN BATTLE.

### A FRENCH PRACTITIONER'S EXPERIENCES.

Under the horrors of the modern battlefield a certain number of feeble brains give way, and already many asylums are occupied by unhappy beings whom the war has rendered insane. But in addition to cases of ordinary insanity are to be noted other curious mental conditions, and one of these, produced by the stress of the combat, is a state of hypnosis and somnambulism. An interesting account of this is given by Dr. Milian in the *Paris Médical*. The victim when brought to the hospital appears to be in a condition midway between coma and sleep. He lies on his back immobile in bed, the eyes closed or half-closed, or sometimes wide open, but always fixed and without any movement of the eyelids; and no noise or question or other external stimulation succeeds in arousing him. Even when flies settle on the eyeballs there is no closure of the lids, though the corneal reflex, to ordinary stimulation, usually remains intact; equally the various cutaneous reflexes and the tendon jerks are retained. If the arm is raised it falls, though less heavily than in apoplexy, and there is never the least suggestion of catalepsy. There is no stertorous respiration as in coma, and the face is calm and expressionless, not red and swollen. On the other hand, the patient is not in an ordinary sleep, for he cannot be aroused. In short, the state is one of hypnosis comparable to that produced in hysterical patients by suggestion or by pressure on the eyeballs. Again, if the patient is lifted out of bed and put on his feet he remains standing, and makes no effort either to walk or to get into bed again. Gradually his legs give way, and he sinks down to the ground, but he never falls suddenly so as to hurt himself. If he is led by the hand or pushed from behind he advances slowly, dragging his limbs; or he may be made to walk a few steps by himself, but he soon stops, and if he meets some obstacle, a wall for example, he remains in contact with it and without stirring. Occasionally he moves his hands as though to draw his bayonet from his belt. It is necessary to feed him like a child, but he swallows without difficulty any liquids put into his mouth. A striking feature is the nearly complete mutism which he maintains: he either takes no notice of any question addressed to him or repeats again and again one and the same word, or some unintelligible sound. In some instances very lively emotional disturbances are aroused—tears, an expression of fright—when the patient is questioned in reference to the battle, though to all other topics he remains impassive. With all this the examination of the nervous system yields negative results, and the cerebro-spinal fluid is normal. This state of hypnosis may last for a few days only, or it may be continued for two to three weeks. As it gradually yields the patient begins to feed himself, to rise from his bed to attend to the calls of nature, and in a more or less automatic fashion he may be persuaded to do some simple work, such as sweeping the ward, and so on. Only in the last place, and generally somewhat suddenly, does full consciousness return, and this often under the influence of a comrade who speaks to the patient of his family, native place, or other subject of personal interest.

The victims of this condition are always young men of from twenty to twenty-two years; townsmen rather than countrymen are affected, and the educated and sensitive rather than those of a low standard of culture; in some there have been evidences of hereditary syphilis. Fatigue and insufficient food are important predisposing influences. Hence many cases were seen in the early days of the war, when the fighting was almost continuous, and there was no opportunity for proper meals. The determining cause is sometimes a physical injury, such as a fall into a trench or the stunning influence of a shell explosion; in other instances the exciting influence is a psychical one, as, for example, the recognition of a brother or friend among the dead, the sight of a comrade killed at the victim's side, or a succession of other misfortunes or horrors.

The condition is distinguished from coma by the absence of stertor and of any conjugate deviation of the head and eyes; the expression is calm, and after a time evidences of reviving consciousness are apparent. It is not unlike the state which follows a relatively slight traumatism of the frontal lobes, where there is no injury of a vital part of the brain and no paralytic phenomena. These latter patients regain consciousness in the course of a few days, and have no recollection of the events which have followed the injury. The hypnosis is not simulated, since the clinical picture is reproduced exactly in soldiers who are complete strangers to one another and belonging to different regiments which have never been associated. Further, the patients, so soon as they are cured, are anxious to return to the Front.

